# Supplementary figures and images for: Vertical Transmission at the Pathogen-Symbiont Interface: Serratia symbiotica and Aphids
Source: mBio. 2021 Apr 20;12(2):e00359-21. doi: 10.1128/mBio.00359-21 (PMC8092240; doi:10.1128/mBio.00359-21)

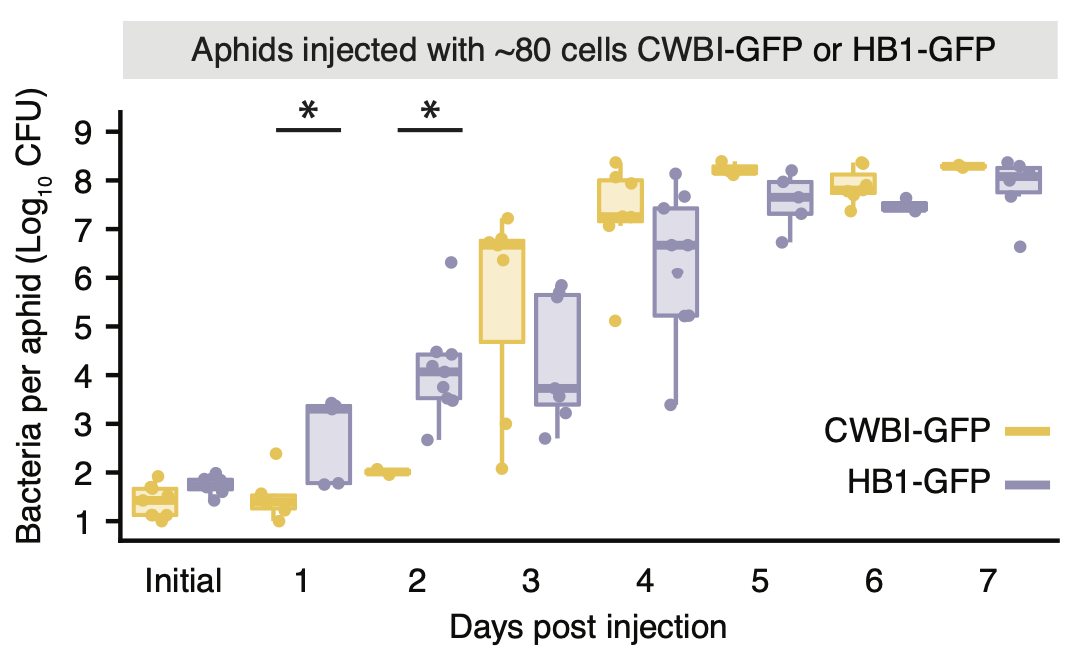

Supplement: FIG S2 [file mBio.00359-21-sf002.tif]
